# *N*-(Ferrocenylmethyl)­dodecan-1-amine

**DOI:** 10.1107/S1600536810005155

**Published:** 2010-02-13

**Authors:** Li-Zhen Sun, Wei An, Hua-Cheng Zhang, Fei-Fei Xin, Ai-You Hao

**Affiliations:** a School of Chemistry and Chemical Engineering, Shandong University, Jinan 250100, People’s Republic of China

## Abstract

The title compound, [Fe(C_5_H_5_)(C_18_H_32_N)], was synthesized by the amination of ferrocenecarbaldehyde. In the complex, the two cyclo­penta­dienyl (Cp) rings are almost parallel with a dihedral angle of 1.36 (8)°, and are separated by a centroid–centroid distance of 3.299 (2) Å. In the crystal, adjacent mol­ecules are linked into a one-dimensional supra­molecular structure *via* weak C—H⋯π inter­actions between the Cp ring H atom and the Cp ring.

## Related literature

For the applications of ferrocene in drug design, see: Atteke *et al.* (2003 ▶); Baramee *et al.* (2006 ▶). For linear ferrocene compounds in supra­molecular chemisty, see: Zhang *et al.* (2010 ▶). For a related structure, see: Zheng & Liu (2009 ▶).
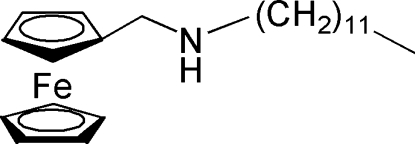


## Experimental

### 

#### Crystal data

[Fe(C_5_H_5_)(C_18_H_32_N)]
*M* *_r_* = 383.39
Monoclinic, 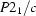

*a* = 26.7005 (6) Å
*b* = 8.0549 (2) Å
*c* = 10.0069 (2) Å
β = 97.534 (1)°
*V* = 2133.60 (8) Å^3^
*Z* = 4
Mo *K*α radiation
μ = 0.71 mm^−1^
*T* = 293 K
0.30 × 0.26 × 0.18 mm

#### Data collection

Bruker APEXII CCD diffractometer
Absorption correction: multi-scan (*SADABS*; Sheldrick, 1996 ▶) *T*_min_ = 0.815, *T*_max_ = 0.881
20623 measured reflections
4900 independent reflections
3892 reflections with *I* > 2σ(*I*)
*R*_int_ = 0.021

#### Refinement

*R*[*F* ^2^ > 2σ(*F* ^2^)] = 0.037
*wR*(*F* ^2^) = 0.112
*S* = 1.09
4900 reflections
228 parameters
H-atom parameters constrained
Δρ_max_ = 0.44 e Å^−3^
Δρ_min_ = −0.37 e Å^−3^

### 

Data collection: *APEX2* (Bruker, 2007 ▶); cell refinement: *SAINT-Plus* (Bruker, 2007 ▶); data reduction: *SAINT-Plus*; program(s) used to solve structure: *SIR97* (Altomare *et al.*, 1999 ▶); program(s) used to refine structure: *SHELXL97* (Sheldrick, 2008 ▶); molecular graphics: *SHELXTL* (Sheldrick, 2008 ▶) and *DIAMOND* (Brandenburg, 1999 ▶); software used to prepare material for publication: *WinGX* (Farrugia, 1999 ▶).

## Supplementary Material

Crystal structure: contains datablocks global, I. DOI: 10.1107/S1600536810005155/hy2280sup1.cif

Structure factors: contains datablocks I. DOI: 10.1107/S1600536810005155/hy2280Isup2.hkl

Additional supplementary materials:  crystallographic information; 3D view; checkCIF report

## Figures and Tables

**Table 1:** Selected bond lengths (Å)

C14—Fe1	2.0502 (16)
C15—Fe1	2.0501 (18)
C16—Fe1	2.038 (2)
C17—Fe1	2.0295 (18)
C18—Fe1	2.0398 (18)
C19—Fe1	2.038 (2)
C20—Fe1	2.0350 (17)
C21—Fe1	2.0375 (18)
C22—Fe1	2.0398 (18)
C23—Fe1	2.0347 (19)

**Table 2:** Hydrogen-bond geometry (Å, °) *Cg*1 is the centroid of the C19–C23 ring.

*D*—H⋯*A*	*D*—H	H⋯*A*	*D*⋯*A*	*D*—H⋯*A*
C18—H18⋯*Cg*1^i^	0.98	2.96	3.870 (2)	155

Symmetry code: (i) 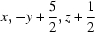.

**Fig. 1.**
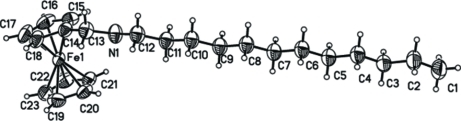
Molecular structure of the title compound. Displacement ellipsoids are drawn at the 50% probability level.

**Fig. 2.**
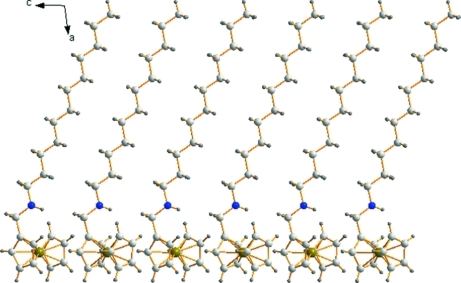
A packing diagram of the title compound along the b axis.

## supplementary crystallographic information

### Comment

The high lipophilicity and electrochemical behaviour of ferrocene render it very attractive for drug design. The incorporation of a ferrocenyl moiety into the 'standard' drug offers new possibilities in therapeutic applications and reversal of drug resistances (Atteke *et al.*, 2003; Baramee *et al.*, 2006). On the other hand, the linear compounds derived from ferrocene have shown great useful functions in supramolecular chemisty (Zhang *et al.*, 2010). In this paper, we report the synthesis and crystal structure of the title compound.

As shown in Fig. 1, the title compound has a tadpole-typed molecular configuration. The planes of two cyclopentadienyl (Cp) rings in the ferrocene scaffold are approximately parallel with a centroid–centroid distance of 3.299 (2) Å and a dihedral angle of 1.36 (8)°. The Fe—C bond distances are in the range of 2.0295 (18) to 2.0502 (16) Å (Table 1), which are consistent with those data in the similar compounds previously reported (Zheng & Liu, 2009). The adjacent molecules are linked by weak C—H···π interactions between ferrocene cores (Table 2), forming a one-dimensional supramolecular architecture as shown in Fig. 2.

### Experimental

A mixture of ferrocenecarbaldehyde (2.415 g, 0.01 mol) and lauryl amine (0.08 mol) in anhydrous methanol (50 ml) was stirred and kept refluxing for 12 h under a nitrogen atmosphere in dark place. NaBH_4_ (0.110 g, 2.9 mmol) was added in small portions. The mixture was stirred for 15 h, and 10 ml acetone was added to stop the reaction. The mixture was extracted with CH_2_Cl_2_ (30 ml) 10 min later. The combined organic phase was dried with anhydrous Na_2_SO_4_, and then evaporated in vacuo. The residue was purified by silica gel column chromatography (eluent, petroleum ether : ethyl acetate = 5:1 by volume) to give the title compound as yellow powder (yield 1.545 g, 80.7%). Single crystals were grown in a mixed solution of hexane/ethyl acetate (2:1 by volume) at room temperature.

### Refinement

H atoms were positioned geometrically and refined as riding atoms, with C—H = 0.98 (CH), 0.97 (CH_2_) and 0.96 (CH_3_) Å and N—H = 0.86 Å and with *U*_iso_(H) = 1.2(1.5 for methyl)*U*_eq_(C,N).

### Crystal data

**Table:**

[Fe(C_5_H_5_)(C_18_H_32_N)]	*F*(000) = 832
*M**_r_* = 383.39	*D*_x_ = 1.194 Mg m^−^^3^
Monoclinic, *P*2_1_/*c*	Mo *K*α radiation, λ = 0.71073 Å
Hall symbol: -P 2ybc	Cell parameters from 8358 reflections
*a* = 26.7005 (6) Å	θ = 2.3–27.4°
*b* = 8.0549 (2) Å	µ = 0.71 mm^−^^1^
*c* = 10.0069 (2) Å	*T* = 293 K
β = 97.534 (1)°	Prism, orange
*V* = 2133.60 (8) Å^3^	0.30 × 0.26 × 0.18 mm
*Z* = 4	

### Data collection

**Table:**

Bruker APEXII CCD diffractometer	4900 independent reflections
Radiation source: fine-focus sealed tube	3892 reflections with *I* > 2σ(*I*)
graphite	*R*_int_ = 0.021
φ and ω scans	θ_max_ = 27.5°, θ_min_ = 0.8°
Absorption correction: multi-scan (*SADABS*; Sheldrick, 1996)	*h* = −34→34
*T*_min_ = 0.815, *T*_max_ = 0.881	*k* = −10→9
20623 measured reflections	*l* = −12→11

### Refinement

**Table:**

Refinement on *F*^2^	Secondary atom site location: difference Fourier map
Least-squares matrix: full	Hydrogen site location: inferred from neighbouring sites
*R*[*F*^2^ > 2σ(*F*^2^)] = 0.037	H-atom parameters constrained
*wR*(*F*^2^) = 0.112	*w* = 1/[σ^2^(*F*_o_^2^) + (0.0649*P*)^2^ + 0.2305*P*] where *P* = (*F*_o_^2^ + 2*F*_c_^2^)/3
*S* = 1.09	(Δ/σ)_max_ = 0.002
4900 reflections	Δρ_max_ = 0.44 e Å^−^^3^
228 parameters	Δρ_min_ = −0.37 e Å^−^^3^
0 restraints	Extinction correction: *SHELXL*, Fc^*^=kFc[1+0.001xFc^2^λ^3^/sin(2θ)]^-1/4^
Primary atom site location: structure-invariant direct methods	Extinction coefficient: 0.0051 (7)

### Fractional atomic coordinates and isotropic or equivalent isotropic displacement parameters (Å^2^)

**Table:**

	*x*	*y*	*z*	*U*_iso_*/*U*_eq_	
C1	−0.25735 (8)	0.8925 (3)	0.3331 (2)	0.0855 (7)	
H1A	−0.2495	0.9931	0.2887	0.128*	
H1B	−0.2924	0.8926	0.3453	0.128*	
H1C	−0.2505	0.7988	0.2791	0.128*	
C2	−0.22527 (7)	0.8816 (3)	0.4689 (2)	0.0688 (6)	
H2A	−0.2342	0.9724	0.5248	0.083*	
H2B	−0.2331	0.7787	0.5121	0.083*	
C3	−0.16928 (7)	0.8879 (3)	0.46240 (19)	0.0569 (4)	
H3A	−0.1616	0.9896	0.4174	0.068*	
H3B	−0.1603	0.7958	0.4078	0.068*	
C4	−0.13669 (6)	0.8803 (3)	0.59851 (18)	0.0570 (5)	
H4A	−0.1452	0.9737	0.6525	0.068*	
H4B	−0.1448	0.7796	0.6443	0.068*	
C5	−0.08054 (6)	0.8833 (3)	0.59124 (19)	0.0558 (4)	
H5A	−0.0726	0.9823	0.5429	0.067*	
H5B	−0.0719	0.7879	0.5397	0.067*	
C6	−0.04805 (6)	0.8813 (3)	0.72736 (18)	0.0558 (4)	
H6A	−0.0564	0.9776	0.7783	0.067*	
H6B	−0.0564	0.7832	0.7762	0.067*	
C7	0.00828 (6)	0.8818 (3)	0.72004 (18)	0.0557 (4)	
H7A	0.0165	0.9785	0.6695	0.067*	
H7B	0.0167	0.7842	0.6708	0.067*	
C8	0.04069 (6)	0.8834 (3)	0.85633 (19)	0.0555 (4)	
H8A	0.0319	0.7879	0.9077	0.067*	
H8B	0.0328	0.9823	0.9048	0.067*	
C9	0.09721 (6)	0.8801 (3)	0.84908 (18)	0.0555 (4)	
H9A	0.1052	0.7802	0.8020	0.067*	
H9B	0.1059	0.9744	0.7964	0.067*	
C10	0.12966 (6)	0.8847 (3)	0.98511 (18)	0.0546 (4)	
H10A	0.1202	0.7927	1.0391	0.066*	
H10B	0.1227	0.9866	1.0308	0.066*	
C11	0.18590 (6)	0.8749 (3)	0.97731 (18)	0.0558 (4)	
H11A	0.1930	0.7717	0.9335	0.067*	
H11B	0.1953	0.9653	0.9216	0.067*	
C12	0.21815 (6)	0.8834 (3)	1.11265 (18)	0.0558 (4)	
H12A	0.2132	0.9901	1.1540	0.067*	
H12B	0.2076	0.7976	1.1709	0.067*	
C13	0.30453 (6)	0.8840 (3)	1.22683 (17)	0.0544 (4)	
H13A	0.2953	0.8050	1.2925	0.065*	
H13B	0.2998	0.9948	1.2611	0.065*	
C14	0.35911 (6)	0.8607 (2)	1.21065 (17)	0.0483 (4)	
C15	0.37909 (7)	0.7502 (2)	1.12038 (19)	0.0560 (4)	
H15	0.3594	0.6787	1.0537	0.067*	
C16	0.43262 (8)	0.7632 (3)	1.1426 (2)	0.0698 (6)	
H16	0.4563	0.7016	1.0943	0.084*	
C17	0.44560 (7)	0.8800 (3)	1.2456 (2)	0.0731 (6)	
H17	0.4800	0.9137	1.2817	0.088*	
C18	0.40041 (7)	0.9412 (3)	1.28863 (18)	0.0591 (5)	
H18	0.3981	1.0241	1.3593	0.071*	
C19	0.39885 (9)	1.2394 (3)	1.0622 (2)	0.0687 (6)	
H19	0.3971	1.3232	1.1325	0.082*	
C20	0.35736 (7)	1.1625 (2)	0.98373 (19)	0.0597 (5)	
H20	0.3216	1.1831	0.9899	0.072*	
C21	0.37662 (8)	1.0532 (3)	0.89473 (19)	0.0596 (5)	
H21	0.3566	0.9829	0.8279	0.071*	
C22	0.42930 (8)	1.0612 (3)	0.9174 (2)	0.0639 (5)	
H22	0.4525	0.9971	0.8693	0.077*	
C23	0.44322 (7)	1.1742 (3)	1.0199 (2)	0.0680 (6)	
H23	0.4779	1.2043	1.0563	0.082*	
Fe1	0.401688 (8)	0.98868 (3)	1.08888 (2)	0.04257 (11)	
N1	0.27153 (5)	0.8617 (2)	1.10053 (15)	0.0577 (4)	
H1	0.2825	0.8379	1.0258	0.069*	

### Atomic displacement parameters (Å^2^)

**Table:**

	*U*^11^	*U*^22^	*U*^33^	*U*^12^	*U*^13^	*U*^23^
C1	0.0610 (13)	0.1023 (19)	0.0867 (16)	−0.0100 (12)	−0.0147 (12)	0.0247 (14)
C2	0.0478 (10)	0.0909 (16)	0.0660 (12)	−0.0064 (10)	0.0012 (9)	0.0139 (11)
C3	0.0481 (10)	0.0660 (11)	0.0557 (11)	−0.0037 (8)	0.0038 (8)	0.0017 (9)
C4	0.0462 (9)	0.0722 (12)	0.0524 (10)	−0.0024 (8)	0.0063 (8)	0.0028 (9)
C5	0.0452 (9)	0.0680 (12)	0.0538 (10)	−0.0006 (8)	0.0057 (8)	−0.0001 (9)
C6	0.0442 (9)	0.0686 (12)	0.0546 (10)	−0.0006 (8)	0.0069 (8)	0.0008 (9)
C7	0.0444 (9)	0.0677 (12)	0.0549 (10)	−0.0004 (8)	0.0063 (8)	−0.0007 (9)
C8	0.0439 (9)	0.0677 (12)	0.0552 (10)	−0.0006 (8)	0.0081 (8)	−0.0008 (9)
C9	0.0444 (9)	0.0674 (12)	0.0553 (11)	−0.0009 (8)	0.0090 (8)	−0.0011 (9)
C10	0.0421 (9)	0.0679 (12)	0.0541 (10)	0.0003 (8)	0.0076 (8)	−0.0011 (9)
C11	0.0430 (9)	0.0709 (12)	0.0542 (10)	−0.0015 (8)	0.0085 (8)	0.0015 (9)
C12	0.0408 (9)	0.0704 (12)	0.0571 (11)	−0.0005 (8)	0.0098 (8)	−0.0004 (9)
C13	0.0451 (9)	0.0702 (12)	0.0488 (10)	−0.0006 (8)	0.0099 (8)	0.0018 (8)
C14	0.0450 (9)	0.0577 (10)	0.0422 (8)	0.0011 (8)	0.0057 (7)	0.0087 (7)
C15	0.0598 (11)	0.0504 (10)	0.0601 (11)	0.0051 (8)	0.0169 (9)	0.0106 (8)
C16	0.0587 (12)	0.0736 (13)	0.0800 (15)	0.0258 (10)	0.0200 (10)	0.0332 (12)
C17	0.0439 (10)	0.1064 (18)	0.0655 (13)	0.0029 (11)	−0.0057 (9)	0.0377 (13)
C18	0.0569 (11)	0.0795 (12)	0.0391 (9)	−0.0042 (10)	−0.0007 (8)	0.0119 (9)
C19	0.1042 (18)	0.0467 (10)	0.0548 (11)	0.0008 (10)	0.0089 (11)	−0.0005 (8)
C20	0.0475 (9)	0.0644 (11)	0.0671 (12)	0.0135 (9)	0.0077 (9)	0.0209 (10)
C21	0.0716 (13)	0.0614 (11)	0.0421 (9)	0.0004 (10)	−0.0060 (9)	0.0084 (9)
C22	0.0663 (12)	0.0721 (12)	0.0580 (12)	0.0155 (11)	0.0253 (10)	0.0193 (10)
C23	0.0512 (10)	0.0744 (13)	0.0760 (14)	−0.0130 (10)	−0.0002 (10)	0.0244 (11)
Fe1	0.03650 (15)	0.05241 (17)	0.03810 (16)	0.00326 (9)	0.00232 (10)	0.00551 (9)
N1	0.0407 (7)	0.0843 (11)	0.0486 (8)	−0.0016 (7)	0.0081 (6)	−0.0071 (8)

### Geometric parameters (Å, °)

**Table:**

C1—C2	1.511 (3)	C12—H12B	0.9700
C1—H1A	0.9600	C13—N1	1.454 (2)
C1—H1B	0.9600	C13—C14	1.499 (2)
C1—H1C	0.9600	C13—H13A	0.9700
C2—C3	1.506 (2)	C13—H13B	0.9700
C2—H2A	0.9700	C14—C15	1.421 (2)
C2—H2B	0.9700	C14—C18	1.421 (3)
C3—C4	1.518 (2)	C14—Fe1	2.0502 (16)
C3—H3A	0.9700	C15—C16	1.421 (3)
C3—H3B	0.9700	C15—Fe1	2.0501 (18)
C4—C5	1.511 (2)	C15—H15	0.9800
C4—H4A	0.9700	C16—C17	1.406 (3)
C4—H4B	0.9700	C16—Fe1	2.038 (2)
C5—C6	1.516 (2)	C16—H16	0.9800
C5—H5A	0.9700	C17—C18	1.421 (3)
C5—H5B	0.9700	C17—Fe1	2.0295 (18)
C6—C7	1.515 (2)	C17—H17	0.9800
C6—H6A	0.9700	C18—Fe1	2.0398 (18)
C6—H6B	0.9700	C18—H18	0.9800
C7—C8	1.516 (2)	C19—C23	1.411 (3)
C7—H7A	0.9700	C19—C20	1.414 (3)
C7—H7B	0.9700	C19—Fe1	2.038 (2)
C8—C9	1.520 (2)	C19—H19	0.9800
C8—H8A	0.9700	C20—C21	1.398 (3)
C8—H8B	0.9700	C20—Fe1	2.0350 (17)
C9—C10	1.515 (2)	C20—H20	0.9800
C9—H9A	0.9700	C21—C22	1.397 (3)
C9—H9B	0.9700	C21—Fe1	2.0375 (18)
C10—C11	1.516 (2)	C21—H21	0.9800
C10—H10A	0.9700	C22—C23	1.386 (3)
C10—H10B	0.9700	C22—Fe1	2.0398 (18)
C11—C12	1.508 (2)	C22—H22	0.9800
C11—H11A	0.9700	C23—Fe1	2.0347 (19)
C11—H11B	0.9700	C23—H23	0.9800
C12—N1	1.457 (2)	N1—H1	0.8600
C12—H12A	0.9700		
			
C2—C1—H1A	109.5	C17—C16—H16	126.0
C2—C1—H1B	109.5	C15—C16—H16	126.0
H1A—C1—H1B	109.5	Fe1—C16—H16	126.0
C2—C1—H1C	109.5	C16—C17—C18	108.48 (19)
H1A—C1—H1C	109.5	C16—C17—Fe1	70.11 (11)
H1B—C1—H1C	109.5	C18—C17—Fe1	69.95 (10)
C3—C2—C1	114.09 (18)	C16—C17—H17	125.8
C3—C2—H2A	108.7	C18—C17—H17	125.8
C1—C2—H2A	108.7	Fe1—C17—H17	125.8
C3—C2—H2B	108.7	C17—C18—C14	107.7 (2)
C1—C2—H2B	108.7	C17—C18—Fe1	69.17 (11)
H2A—C2—H2B	107.6	C14—C18—Fe1	70.06 (10)
C2—C3—C4	114.54 (16)	C17—C18—H18	126.2
C2—C3—H3A	108.6	C14—C18—H18	126.2
C4—C3—H3A	108.6	Fe1—C18—H18	126.2
C2—C3—H3B	108.6	C23—C19—C20	107.33 (19)
C4—C3—H3B	108.6	C23—C19—Fe1	69.62 (12)
H3A—C3—H3B	107.6	C20—C19—Fe1	69.58 (11)
C5—C4—C3	114.32 (15)	C23—C19—H19	126.3
C5—C4—H4A	108.7	C20—C19—H19	126.3
C3—C4—H4A	108.7	Fe1—C19—H19	126.3
C5—C4—H4B	108.7	C21—C20—C19	107.65 (17)
C3—C4—H4B	108.7	C21—C20—Fe1	70.03 (10)
H4A—C4—H4B	107.6	C19—C20—Fe1	69.78 (11)
C4—C5—C6	114.27 (15)	C21—C20—H20	126.2
C4—C5—H5A	108.7	C19—C20—H20	126.2
C6—C5—H5A	108.7	Fe1—C20—H20	126.2
C4—C5—H5B	108.7	C22—C21—C20	108.31 (19)
C6—C5—H5B	108.7	C22—C21—Fe1	70.06 (11)
H5A—C5—H5B	107.6	C20—C21—Fe1	69.83 (10)
C7—C6—C5	114.28 (15)	C22—C21—H21	125.8
C7—C6—H6A	108.7	C20—C21—H21	125.8
C5—C6—H6A	108.7	Fe1—C21—H21	125.8
C7—C6—H6B	108.7	C23—C22—C21	108.50 (19)
C5—C6—H6B	108.7	C23—C22—Fe1	69.92 (11)
H6A—C6—H6B	107.6	C21—C22—Fe1	69.88 (11)
C6—C7—C8	114.18 (15)	C23—C22—H22	125.7
C6—C7—H7A	108.7	C21—C22—H22	125.7
C8—C7—H7A	108.7	Fe1—C22—H22	125.7
C6—C7—H7B	108.7	C22—C23—C19	108.21 (19)
C8—C7—H7B	108.7	C22—C23—Fe1	70.32 (11)
H7A—C7—H7B	107.6	C19—C23—Fe1	69.84 (11)
C7—C8—C9	114.20 (15)	C22—C23—H23	125.9
C7—C8—H8A	108.7	C19—C23—H23	125.9
C9—C8—H8A	108.7	Fe1—C23—H23	125.9
C7—C8—H8B	108.7	C17—Fe1—C23	107.07 (9)
C9—C8—H8B	108.7	C17—Fe1—C20	159.10 (10)
H8A—C8—H8B	107.6	C23—Fe1—C20	67.99 (8)
C10—C9—C8	114.26 (15)	C17—Fe1—C21	158.62 (10)
C10—C9—H9A	108.7	C23—Fe1—C21	67.35 (9)
C8—C9—H9A	108.7	C20—Fe1—C21	40.14 (8)
C10—C9—H9B	108.7	C17—Fe1—C19	122.45 (10)
C8—C9—H9B	108.7	C23—Fe1—C19	40.54 (8)
H9A—C9—H9B	107.6	C20—Fe1—C19	40.63 (8)
C9—C10—C11	113.96 (15)	C21—Fe1—C19	67.69 (9)
C9—C10—H10A	108.8	C17—Fe1—C16	40.44 (9)
C11—C10—H10A	108.8	C23—Fe1—C16	121.59 (8)
C9—C10—H10B	108.8	C20—Fe1—C16	159.64 (10)
C11—C10—H10B	108.8	C21—Fe1—C16	123.34 (10)
H10A—C10—H10B	107.7	C19—Fe1—C16	157.65 (9)
C12—C11—C10	113.83 (15)	C17—Fe1—C18	40.89 (8)
C12—C11—H11A	108.8	C23—Fe1—C18	123.36 (9)
C10—C11—H11A	108.8	C20—Fe1—C18	123.53 (8)
C12—C11—H11B	108.8	C21—Fe1—C18	159.49 (9)
C10—C11—H11B	108.8	C19—Fe1—C18	108.01 (9)
H11A—C11—H11B	107.7	C16—Fe1—C18	68.47 (9)
N1—C12—C11	111.68 (14)	C17—Fe1—C22	122.53 (8)
N1—C12—H12A	109.3	C23—Fe1—C22	39.76 (9)
C11—C12—H12A	109.3	C20—Fe1—C22	67.54 (8)
N1—C12—H12B	109.3	C21—Fe1—C22	40.06 (8)
C11—C12—H12B	109.3	C19—Fe1—C22	67.50 (9)
H12A—C12—H12B	107.9	C16—Fe1—C22	107.34 (9)
N1—C13—C14	112.18 (14)	C18—Fe1—C22	158.80 (9)
N1—C13—H13A	109.2	C17—Fe1—C15	68.22 (9)
C14—C13—H13A	109.2	C23—Fe1—C15	157.63 (9)
N1—C13—H13B	109.2	C20—Fe1—C15	124.14 (8)
C14—C13—H13B	109.2	C21—Fe1—C15	108.60 (9)
H13A—C13—H13B	107.9	C19—Fe1—C15	160.45 (9)
C15—C14—C18	107.81 (16)	C16—Fe1—C15	40.68 (8)
C15—C14—C13	126.77 (17)	C18—Fe1—C15	68.33 (9)
C18—C14—C13	125.36 (17)	C22—Fe1—C15	122.93 (9)
C15—C14—Fe1	69.72 (10)	C17—Fe1—C14	68.47 (7)
C18—C14—Fe1	69.27 (10)	C23—Fe1—C14	160.09 (9)
C13—C14—Fe1	128.60 (12)	C20—Fe1—C14	108.84 (7)
C14—C15—C16	107.96 (18)	C21—Fe1—C14	123.81 (8)
C14—C15—Fe1	69.72 (10)	C19—Fe1—C14	124.22 (8)
C16—C15—Fe1	69.20 (11)	C16—Fe1—C14	68.43 (7)
C14—C15—H15	126.0	C18—Fe1—C14	40.67 (7)
C16—C15—H15	126.0	C22—Fe1—C14	159.01 (9)
Fe1—C15—H15	126.0	C15—Fe1—C14	40.56 (7)
C17—C16—C15	108.06 (18)	C13—N1—C12	113.63 (14)
C17—C16—Fe1	69.45 (12)	C13—N1—H1	123.2
C15—C16—Fe1	70.12 (10)	C12—N1—H1	123.2
			
C1—C2—C3—C4	178.87 (19)	C22—C21—Fe1—C14	−161.73 (13)
C2—C3—C4—C5	178.88 (17)	C20—C21—Fe1—C14	78.98 (14)
C3—C4—C5—C6	178.17 (17)	C23—C19—Fe1—C17	77.97 (15)
C4—C5—C6—C7	179.15 (16)	C20—C19—Fe1—C17	−163.53 (12)
C5—C6—C7—C8	178.77 (16)	C20—C19—Fe1—C23	118.50 (18)
C6—C7—C8—C9	178.73 (16)	C23—C19—Fe1—C20	−118.50 (18)
C7—C8—C9—C10	179.00 (16)	C23—C19—Fe1—C21	−80.76 (14)
C8—C9—C10—C11	177.84 (16)	C20—C19—Fe1—C21	37.75 (12)
C9—C10—C11—C12	178.69 (16)	C23—C19—Fe1—C16	43.6 (3)
C10—C11—C12—N1	175.96 (16)	C20—C19—Fe1—C16	162.1 (2)
N1—C13—C14—C15	−33.2 (2)	C23—C19—Fe1—C18	120.63 (13)
N1—C13—C14—C18	149.91 (17)	C20—C19—Fe1—C18	−120.86 (12)
N1—C13—C14—Fe1	59.3 (2)	C23—C19—Fe1—C22	−37.26 (12)
C18—C14—C15—C16	−0.18 (19)	C20—C19—Fe1—C22	81.24 (13)
C13—C14—C15—C16	−177.53 (16)	C23—C19—Fe1—C15	−163.6 (2)
Fe1—C14—C15—C16	58.82 (12)	C20—C19—Fe1—C15	−45.1 (3)
C18—C14—C15—Fe1	−59.00 (12)	C23—C19—Fe1—C14	162.61 (12)
C13—C14—C15—Fe1	123.65 (17)	C20—C19—Fe1—C14	−78.89 (13)
C14—C15—C16—C17	0.2 (2)	C15—C16—Fe1—C17	119.18 (16)
Fe1—C15—C16—C17	59.31 (14)	C17—C16—Fe1—C23	79.02 (14)
C14—C15—C16—Fe1	−59.14 (12)	C15—C16—Fe1—C23	−161.80 (12)
C15—C16—C17—C18	−0.1 (2)	C17—C16—Fe1—C20	−167.86 (19)
Fe1—C16—C17—C18	59.64 (14)	C15—C16—Fe1—C20	−48.7 (3)
C15—C16—C17—Fe1	−59.72 (12)	C17—C16—Fe1—C21	161.17 (12)
C16—C17—C18—C14	0.0 (2)	C15—C16—Fe1—C21	−79.65 (14)
Fe1—C17—C18—C14	59.71 (13)	C17—C16—Fe1—C19	47.3 (3)
C16—C17—C18—Fe1	−59.74 (14)	C15—C16—Fe1—C19	166.45 (19)
C15—C14—C18—C17	0.1 (2)	C17—C16—Fe1—C18	−37.81 (12)
C13—C14—C18—C17	177.53 (16)	C15—C16—Fe1—C18	81.37 (12)
Fe1—C14—C18—C17	−59.15 (13)	C17—C16—Fe1—C22	120.13 (13)
C15—C14—C18—Fe1	59.28 (12)	C15—C16—Fe1—C22	−120.69 (12)
C13—C14—C18—Fe1	−123.32 (17)	C17—C16—Fe1—C15	−119.18 (17)
C23—C19—C20—C21	−0.4 (2)	C17—C16—Fe1—C14	−81.69 (12)
Fe1—C19—C20—C21	−60.05 (13)	C15—C16—Fe1—C14	37.49 (11)
C23—C19—C20—Fe1	59.65 (14)	C14—C18—Fe1—C17	−118.95 (19)
C19—C20—C21—C22	0.2 (2)	C17—C18—Fe1—C23	−77.08 (17)
Fe1—C20—C21—C22	−59.72 (13)	C14—C18—Fe1—C23	163.97 (11)
C19—C20—C21—Fe1	59.90 (13)	C17—C18—Fe1—C20	−161.22 (14)
C20—C21—C22—C23	0.1 (2)	C14—C18—Fe1—C20	79.83 (14)
Fe1—C21—C22—C23	−59.46 (14)	C17—C18—Fe1—C21	166.6 (2)
C20—C21—C22—Fe1	59.58 (13)	C14—C18—Fe1—C21	47.6 (3)
C21—C22—C23—C19	−0.4 (2)	C17—C18—Fe1—C19	−119.11 (15)
Fe1—C22—C23—C19	−59.81 (14)	C14—C18—Fe1—C19	121.94 (13)
C21—C22—C23—Fe1	59.43 (14)	C17—C18—Fe1—C16	37.41 (14)
C20—C19—C23—C22	0.5 (2)	C14—C18—Fe1—C16	−81.54 (13)
Fe1—C19—C23—C22	60.10 (14)	C17—C18—Fe1—C22	−45.1 (3)
C20—C19—C23—Fe1	−59.62 (14)	C14—C18—Fe1—C22	−164.0 (2)
C16—C17—Fe1—C23	−118.99 (13)	C17—C18—Fe1—C15	81.32 (15)
C18—C17—Fe1—C23	121.61 (14)	C14—C18—Fe1—C15	−37.63 (11)
C16—C17—Fe1—C20	168.17 (19)	C17—C18—Fe1—C14	118.95 (19)
C18—C17—Fe1—C20	48.8 (3)	C23—C22—Fe1—C17	−77.16 (16)
C16—C17—Fe1—C21	−47.7 (3)	C21—C22—Fe1—C17	163.26 (14)
C18—C17—Fe1—C21	−167.1 (2)	C21—C22—Fe1—C23	−119.59 (18)
C16—C17—Fe1—C19	−160.67 (12)	C23—C22—Fe1—C20	82.12 (13)
C18—C17—Fe1—C19	79.94 (15)	C21—C22—Fe1—C20	−37.47 (13)
C18—C17—Fe1—C16	−119.40 (19)	C23—C22—Fe1—C21	119.59 (18)
C16—C17—Fe1—C18	119.40 (19)	C23—C22—Fe1—C19	37.97 (12)
C16—C17—Fe1—C22	−78.27 (15)	C21—C22—Fe1—C19	−81.62 (14)
C18—C17—Fe1—C22	162.33 (14)	C23—C22—Fe1—C16	−118.87 (14)
C16—C17—Fe1—C15	37.80 (12)	C21—C22—Fe1—C16	121.55 (14)
C18—C17—Fe1—C15	−81.60 (14)	C23—C22—Fe1—C18	−43.8 (3)
C16—C17—Fe1—C14	81.59 (12)	C21—C22—Fe1—C18	−163.4 (2)
C18—C17—Fe1—C14	−37.81 (13)	C23—C22—Fe1—C15	−160.77 (12)
C22—C23—Fe1—C17	120.69 (14)	C21—C22—Fe1—C15	79.64 (14)
C19—C23—Fe1—C17	−120.31 (14)	C23—C22—Fe1—C14	166.26 (18)
C22—C23—Fe1—C20	−80.88 (13)	C21—C22—Fe1—C14	46.7 (3)
C19—C23—Fe1—C20	38.12 (12)	C14—C15—Fe1—C17	81.90 (12)
C22—C23—Fe1—C21	−37.33 (12)	C16—C15—Fe1—C17	−37.58 (12)
C19—C23—Fe1—C21	81.67 (13)	C14—C15—Fe1—C23	163.83 (18)
C22—C23—Fe1—C19	−119.00 (18)	C16—C15—Fe1—C23	44.4 (3)
C22—C23—Fe1—C16	78.93 (15)	C14—C15—Fe1—C20	−78.92 (13)
C19—C23—Fe1—C16	−162.07 (13)	C16—C15—Fe1—C20	161.60 (13)
C22—C23—Fe1—C18	162.56 (12)	C14—C15—Fe1—C21	−120.65 (12)
C19—C23—Fe1—C18	−78.44 (15)	C16—C15—Fe1—C21	119.88 (13)
C19—C23—Fe1—C22	119.00 (18)	C14—C15—Fe1—C19	−45.1 (3)
C22—C23—Fe1—C15	46.6 (3)	C16—C15—Fe1—C19	−164.6 (2)
C19—C23—Fe1—C15	165.58 (19)	C14—C15—Fe1—C16	119.48 (16)
C22—C23—Fe1—C14	−165.54 (18)	C14—C15—Fe1—C18	37.73 (11)
C19—C23—Fe1—C14	−46.5 (3)	C16—C15—Fe1—C18	−81.75 (13)
C21—C20—Fe1—C17	160.6 (2)	C14—C15—Fe1—C22	−162.56 (11)
C19—C20—Fe1—C17	42.1 (3)	C16—C15—Fe1—C22	77.96 (14)
C21—C20—Fe1—C23	80.51 (14)	C16—C15—Fe1—C14	−119.48 (16)
C19—C20—Fe1—C23	−38.03 (13)	C15—C14—Fe1—C17	−81.23 (13)
C19—C20—Fe1—C21	−118.53 (17)	C18—C14—Fe1—C17	38.01 (14)
C21—C20—Fe1—C19	118.53 (17)	C13—C14—Fe1—C17	157.32 (19)
C21—C20—Fe1—C16	−41.8 (3)	C15—C14—Fe1—C23	−161.9 (2)
C19—C20—Fe1—C16	−160.4 (2)	C18—C14—Fe1—C23	−42.6 (3)
C21—C20—Fe1—C18	−163.16 (13)	C13—C14—Fe1—C23	76.7 (3)
C19—C20—Fe1—C18	78.31 (14)	C15—C14—Fe1—C20	120.88 (12)
C21—C20—Fe1—C22	37.40 (13)	C18—C14—Fe1—C20	−119.89 (13)
C19—C20—Fe1—C22	−81.13 (14)	C13—C14—Fe1—C20	−0.57 (18)
C21—C20—Fe1—C15	−78.09 (14)	C15—C14—Fe1—C21	78.92 (13)
C19—C20—Fe1—C15	163.37 (11)	C18—C14—Fe1—C21	−161.85 (13)
C21—C20—Fe1—C14	−120.49 (12)	C13—C14—Fe1—C21	−42.53 (19)
C19—C20—Fe1—C14	120.98 (12)	C15—C14—Fe1—C19	163.35 (12)
C22—C21—Fe1—C17	−41.8 (3)	C18—C14—Fe1—C19	−77.42 (14)
C20—C21—Fe1—C17	−161.1 (2)	C13—C14—Fe1—C19	41.90 (19)
C22—C21—Fe1—C23	37.06 (13)	C15—C14—Fe1—C16	−37.61 (12)
C20—C21—Fe1—C23	−82.23 (13)	C18—C14—Fe1—C16	81.63 (14)
C22—C21—Fe1—C20	119.30 (18)	C13—C14—Fe1—C16	−159.05 (19)
C22—C21—Fe1—C19	81.10 (14)	C15—C14—Fe1—C18	−119.24 (16)
C20—C21—Fe1—C19	−38.20 (12)	C13—C14—Fe1—C18	119.3 (2)
C22—C21—Fe1—C16	−76.83 (16)	C15—C14—Fe1—C22	44.6 (2)
C20—C21—Fe1—C16	163.88 (12)	C18—C14—Fe1—C22	163.9 (2)
C22—C21—Fe1—C18	162.9 (2)	C13—C14—Fe1—C22	−76.8 (3)
C20—C21—Fe1—C18	43.6 (3)	C18—C14—Fe1—C15	119.24 (16)
C20—C21—Fe1—C22	−119.30 (18)	C13—C14—Fe1—C15	−121.4 (2)
C22—C21—Fe1—C15	−119.41 (13)	C14—C13—N1—C12	179.95 (15)
C20—C21—Fe1—C15	121.30 (12)	C11—C12—N1—C13	174.70 (16)

### Hydrogen-bond geometry (Å, °)

**Table:**

Cg1 is the centroid of the C19–C23 ring.

**Table:**

*D*—H···*A*	*D*—H	H···*A*	*D*···*A*	*D*—H···*A*
C18—H18···Cg1^i^	0.98	2.96	3.870 (2)	155

Symmetry codes: (i) *x*, −*y*+5/2, *z*+1/2.
